# Characteristics of Cohesin Mutation in Acute Myeloid Leukemia and Its Clinical Significance

**DOI:** 10.3389/fonc.2021.579881

**Published:** 2021-04-13

**Authors:** Caixia Han, Xuefeng Gao, Yonghui Li, Juan Zhang, Erna Yang, Li Zhang, Li Yu

**Affiliations:** Department of Hematology and Oncology, International Cancer Center, Shenzhen Key Laboratory of Precision Medicine for Hematological Malignancies, Shenzhen University General Hospital, Shenzhen University Clinical Medical Academy, Shenzhen University Health Science Center, Shenzhen, China

**Keywords:** cohesin mutation, acute myeloid leukemia, AML1-ETO, hematopoietic stem and progenitor cells, leukemogenesis

## Abstract

The occurrence of gene mutation is a major contributor to the initiation and propagation of acute myeloid leukemia (AML). Accumulating evidence suggests that genes encoding cohesin subunits have a high prevalence of mutations in AML, especially in the t(8;21) subtype. Therefore, it is important to understand how cohesin mutations contribute to leukemogenesis. However, the fundamental understanding of cohesin mutation in clonal expansion and myeloid transformation in hematopoietic cells remains ambiguous. Previous studies briefly introduced the cohesin mutation in AML; however, an in-depth summary of mutations in AML was not provided, and the correlation between cohesin and AML1-ETO in t (8;21) AML was also not analyzed. By summarizing the major findings regarding the cohesin mutation in AML, this review aims to define the characteristics of the cohesin complex mutation, identify its relationships with co-occurring gene mutations, assess its roles in clonal evolution, and discuss its potential for the prognosis of AML. In particular, we focus on the function of cohesin mutations in RUNX1-RUNX1T1 fusion.

## Introduction

It has become apparent that acute myeloid leukemia (AML) is induced by the cooperative action of deregulated genes that alter cell proliferation and differentiation. As compared with other AML cytogenetic groups, patients with t(8; 21) AML had a relatively favorable prognosis, and most of them (>85%) achieved complete remission (CR) with high-dose cytosine arabinoside (Ara-C). However, approximately 30% of the t(8; 21) AML patients relapsed within 1 year, and the overall survival at 5 years was close to 51% (1, 2). Thus, it is imperative to identify the molecular mechanisms that lead to this disease.

Chromosomal translocations and/or gene mutations are the most common chromosome abnormalities in AML. In particular, AML with chromosomal rearrangements t(8;21) (q22;q22) can trigger the generation of the aberrant oncogenic fusion protein AML1-ETO (3, 4). However, AML1-ETO alone is not sufficient to induce the onset of leukemia, and additional genetic/epigenetic abnormalities are required (5, 6). To date, mutations in multiple driver genes have been identified in AML patients with AML1-ETO fusion (7–9), further indicating that AML1-ETO required additional genetic/epigenetic abnormalities to induce t(8;21) leukemogenesis. Recently, investigations, through genome-wide sequencing, revealed recurrent mutations in the cohesin complex genes in AML patients, especially in the t(8;21) subtype (10–13). However, only few studies have investigated the role of cohesin mutation in the pathogenesis of AML.

**Figure 1:**
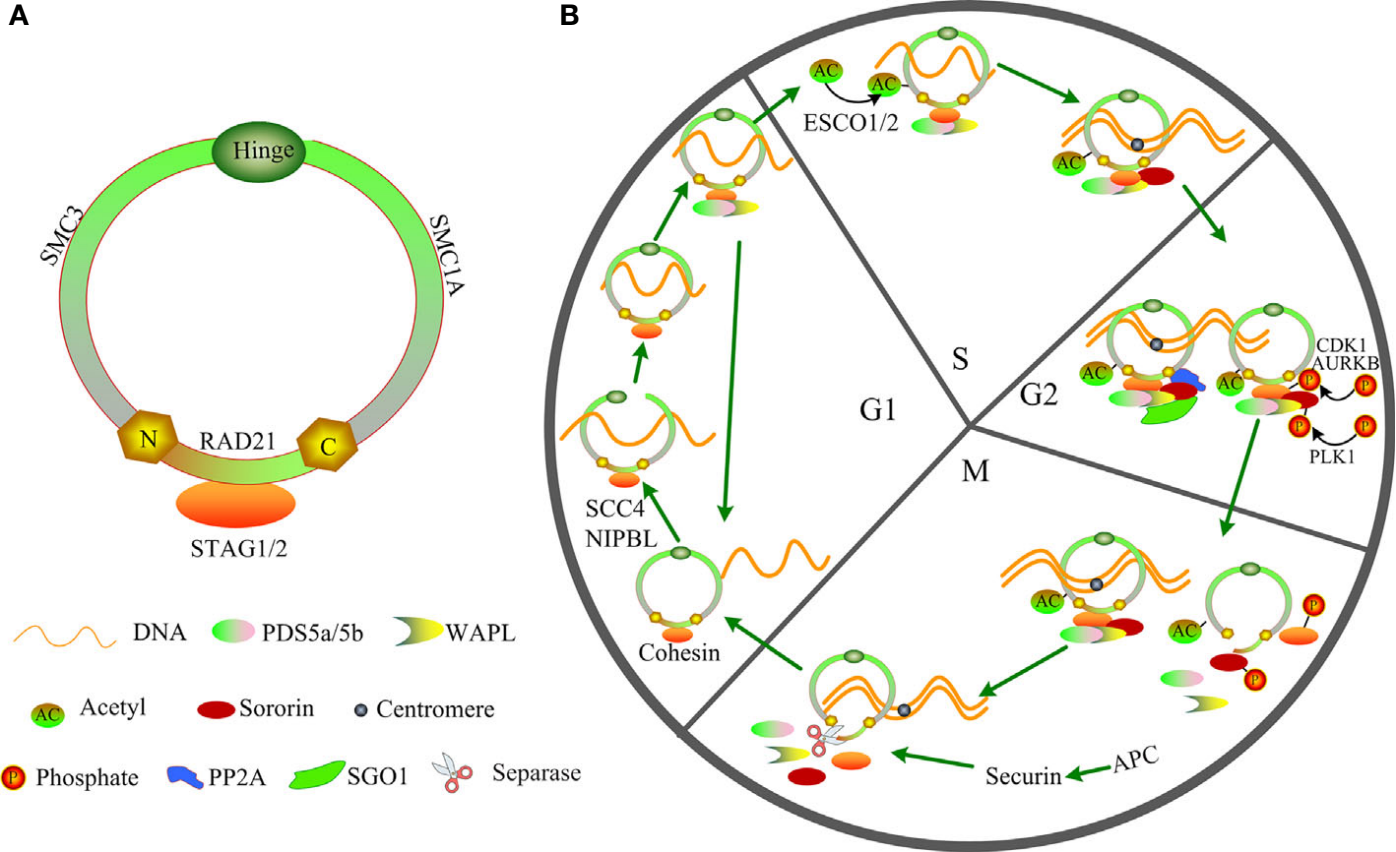
Structure and function of cohesin in the cell cycle. **(A)** Structure of cohesin. **(B)** Function of cohesin in the cell cycle.

Cohesin with a ring shape is composed of the following four core members: structural maintenance of chromosomes (SMC3 and SMC1A), RAD cohesin complex component (RAD21), and cohesin subunit SA (STAG1/STAG2) (**Figure 1A**). During the cell cycle, cohesin facilitates the establishment of cohesion by assisting several additional subunits, including NIPBL, MAU2, WAPL, PDS5A, PDS5B, and sororin (**Figure 1B**) (14–17). Therefore, the ring-shaped cohesin can entrap the sister chromatids, and it regulates the separation of sister chromatids, DNA replication, and repair of the damaged double-strand DNA during cell cycle progression (18–22). Cohesin can exert its functions in increased DNA accessibility for transcription factors (TFs) owing to its cellular memory that promotes re-establishment of TF clusters in the early M phase (23). Cohesin complex can also interact with transcriptional repressor CTCF, promoters, mediators, enhancers, initiation and elongation forms of RNA polymerase II (RNAPII) or TFs to regulate chromatin architecture and gene expression (19, 24–28). Cohesin facilitates the promoter and distant *conserved* regulatory elements (CREs) interactions by “looping out” the intervening chromatin segment to activate or inhibit the gene expression (29, 30). Furthermore, cohesin drives the assembly of the gene enhancer/promoter complex through enhancer-promoter looping, and it produces multiple alternative splicing forms to mediate differential control of space- and stage- specific developmental programs (30–32). Mutation of cohesin can strongly affect the enhancer-promoter interaction (33), therefore altering normal gene expression. Growing evidence indicates that cohesin function deficiency is caused by cohesin mutations (11, 12, 34), suggesting a possible tumor-suppressor function of cohesin in AML. Herein, we review the major findings regarding the cohesin complex mutation, and to discuss the potential molecular functions of cohesin especially in t(8;21) AML by investigating the published references.

## The Characteristics of Cohesin Mutations in AML

The data of cohesin subunits mutations were collected from 16 articles. The percentage of cohesin mutations with different characteristics is displayed in **Table 1**, and detailed data are presented in **Supplement Table 1**.

**Table 1:** Characteristics of AML patients with cohesin mutation.

Variables	STAG2 percentage^◆^	RAD21 percentage^◆^	SMC1A percentage^◆^	SMC3 percentage^◆^	Total percentage^※^
Total	3.93%	3.29%	2.05%	2.56%	11.83%
**Age**					
Pediatric AML	1.44%	4.41%	3.75%	3.03%	12.63%
Adult AML	4.00%	0.98%	2.14%	2.57%	9.69%
**Age for t(8;21) AML**					
Pediatric AML	2.33%	6.94%	6.74%	6.25%	22.26%
Adult AML	2.45%	8.38%	4.91%	3.68%	19.42%
**Gender**					
Male	1.92%	2.88%	2.61%	1.44%	8.85%
Female	1.87%	1.33%	0.27%	3.2%	6.67%
**Chromosomal abnormality**					
t(8;21)	2.4%	8.13%	5.16%	4.18%	19.87%
inv(16)	0	0.25%	0.51%	0.76%	1.52%
11q23	1.18%	1.18%	1.72%	1.18%	5.24%
t(15,17)	0	0	0	0	0
Normal	5.3%	2.27%	3.03%	3.03%	13.60%
Complex	0	0	0	1.49%	1.49%
**Cytogenetic risk^★^**					
Favorable	0	0.10%	2.04%	0.10%	2.24%
Intermediate	2.57%	2.06%	1.54%	2.06%	8.23%
Unfavorable	1.10%	0.00%	1.10%	3.30%	5.49%
**Diagnosis type**					
deno AML	2.18%	2.18%	1.65%	2.47%	8.49%
sAML	6.36%	2.47%	1.06%	2.83%	12.72%
tAML	18.81%	3.96%	2.97%	1.98%	28.71%
**FAB subtype**					
M0	3.23%	0	3.85%	0	7.07%
M1	3.62%	1.45%	3.54%	2.17%	10.79%
M2	2.03%	4.06%	1.44%	4.06%	11.59%
M3	NA	NA	NA	NA	NA
M4	1.59%	1.06%	0.63%	1.59%	4.86%
M5	0	3.97%	1.25%	2.38%	7.60%
M6	15.38%	0	0	0	15.38%
M7	0	0	0	0	0
NA/other	0	0	0	2.33%	2.33%

FAB, French-American-British classification; sAML, secondary AML; tAML, therapy-related AML.

**^★^**The cytogenetic risk group is defined according to Medical Research Council criteria.

^◆^The percentage for each subunit is the number of gene mutation/total samples with genetic testing *100%.

^※^Total percentage is the sum of the percentage of four subunits.

As shown in **Table 1**, cohesin subunit mutations were harbored by a total of 11.88% of AML patients including all cytogenetic groups (**Table 1**). Similar to our study, other studies demonstrated that the most frequently mutated cohesin subunit was STAG2 (3.93%), especially in cytogenetically normal AML (CN-AML) and adult AML (12, 35). Cohesin mutation was found in 19.87% of t(8;21) AML cases, which was much higher than that in the other cytogenetic subgroups, and was rare or none mutation in t(15;17), inv16 and complex chromosomal abnormality groups. Interestingly, the incidence and profile of cohesin gene mutations in t(8;21) AML were different from those in other types of AML (**Table 1**). The frequency of RAD21 mutation (about 8.08%) was higher than the other cohesin compound gene mutations in t(8;21) AML. The mutation frequency of STAG2 (2.4%) was at the bottom of the list for t(8;21) AML. The frequencies of mutations in SMC1A and SMC3 were found to be 5.7% and 4.3%, respectively, in the t(8;21) cases, which were also higher than those in the other AML subtypes. Thus, we posit that RAD21, SMC1A, and SMC3 are more likely to be mutated in t(8;21) AML.

We further analyzed cohesin gene mutation data by comparing between pediatric and adult AML and found that the frequencies of cohesin mutations in childhood AML was higher than adult AML, while RAD21 mutations rarely occurred in adult AML, except for pediatric t(8; 21) AML. STAG2 was the most commonly mutated gene in adult AML. Besides, in agreement with our results, recent studies by Metzeler et al., demonstrated that STAG2 also tended to be more frequent in secondary AML (sAML) compared with *de novo* AML (35). For different FAB types, RAD21 mutations were mainly found in FAB M2 and M5. SMC3 were frequently identified in FAB M2. Because the number of AML patients in FAB M0 and M7 were limited, the proportion of STAG2 and SMC1A mutation was questionable. AML patients with intermediate risk cytogenetics but neither with favorable cytogenetics nor with unfavorable cytogenetics had the highest percentage of STAG2 and RAD21 mutation, and accord with the previous result (11). Intriguingly, the frequency of STAG2, RAD21, and SMC3 mutations in male had little difference from that in female and each type of mutation occur in about 1% to 3% of AML patients. Although SMC1A was located on the X-chromosome, the percentage of SMC1A mutation in male is higher than that in female.

**Figure 2:**
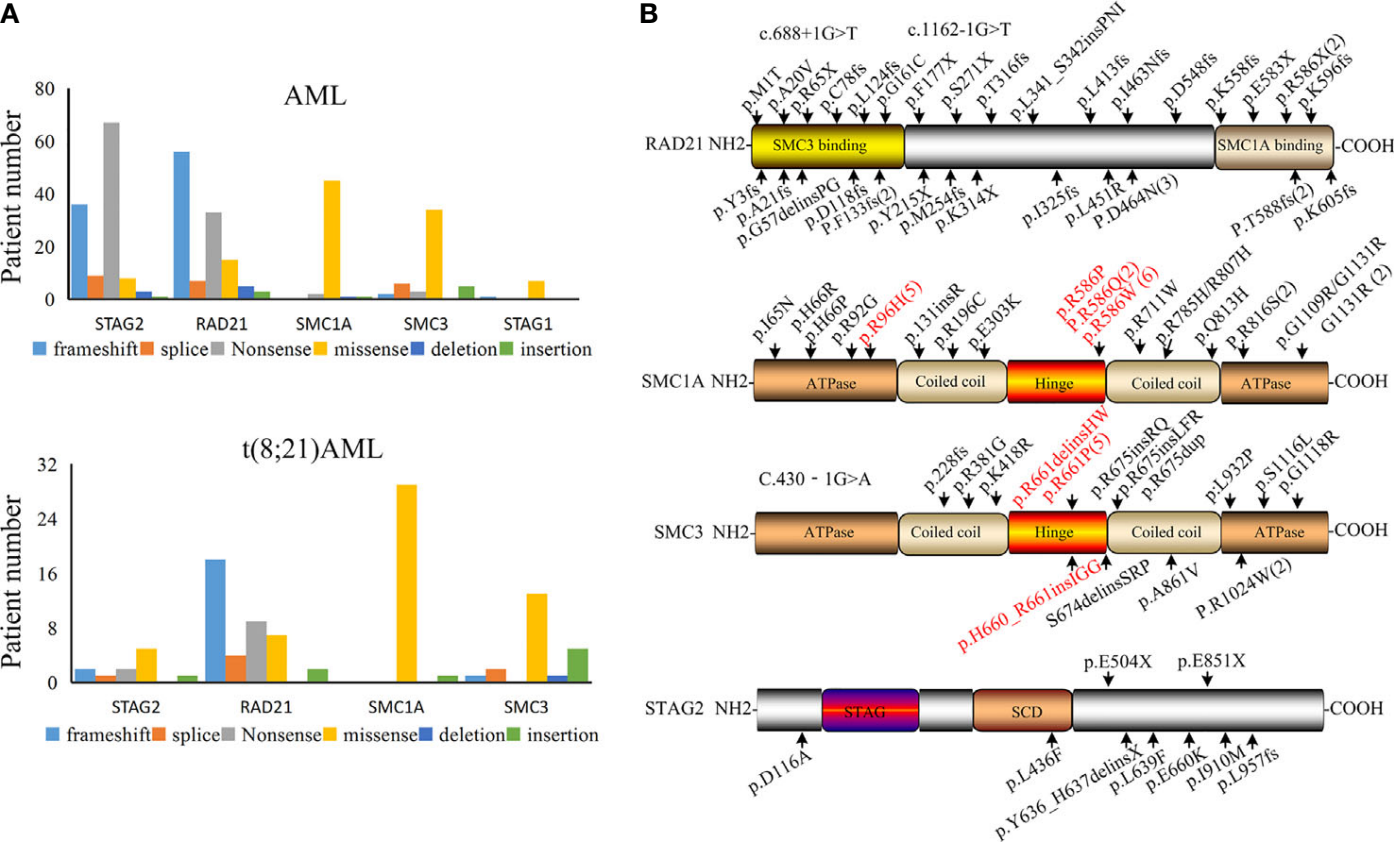
Cohesin mutation in patients with AML. **(A)** Various types of cohesin mutations. **(B)** Location of mutations in the cohesin complex in t(8;21) AML.

The presence of a mutation in cohesin is highly specific for t(8;21) AML, so we next asked whether there were specific mutant form and hotspot in the mutated cohesin gene. We found that the majority of STAG2 and RAD21 mutations were nonsense and frameshift in all the AML subtypes (**Figure 2A** **)**, suggesting that cohesin mutations resulted in a decrease or loss of the cohesin function. Almost all SMC1A and SMC3 mutations were found to be missense, which was consistent with previous studies (35–37).

By summarizing the positions for the identified cohesin mutation genes that reported in t(8;21) AML patients (**Figure 2B**), we found that the mutation sites of RAD21 were present over the whole coding section. A mutated site was found in each domain of the RAD21, but no mutational hotspots were identified. It was found that RAD21 was rapidly cleaved by caspase-3 and -7 at the Asp279 site in apoptosis process (38). RAD21 is not only cleaved at Arg172 and Arg450 sites for sister chromatid separation, but also for the completion of cytokinesis (39). However, no mutations were found in these sites among t(8;21) AML patients based on our analysis, suggesting that the mutated RAD21 might not be involved in apoptosis induced by diverse stimuli or chromatid separation in the development AML1-ETO driven leukemogenesis.

The C-terminal plays an important role in the functions of STAG2 protein, as it is the location of many phosphorylation sites and potentially the nuclear localization signal (40, 41). The majority of STAG2 mutations in t(8;21) AML occur in the C-terminal and potentially result in loss of the phosphorylation site and the nuclear localization signal. These findings may explain why most of the STAG2 mutations were found in the C-terminal of STAG2 in t(8;21) AML patients. It was noted that mutated SMC1A was located at the codons R586 site in the hinge domain among from nine patients and was at the codons R96 site in adenosine triphosphatase heads among five patients in 30 cases with t(8;21) AML. In the data of 38 t(8;21) AML patients, seven mutated SMC3 genes were located at the codons R661 site in the hinge domain (**Figure 2B**). These mutation sites should be considered in functional analyses of these mutations.

## Co-Existing Variants in AML with Cohesin Mutation

Many leukemia genes are found to be infrequently mutated, and AML patients typically have more than one driver mutation (42, 43). Further, cohesin mutations are frequently found with other concurrent gene mutations. The mutational cohesin complex was often to be enriched or mutually antagonistic in specific molecular subgroups of cancer, which implied that mutations in the cohesin gene were more oncogenic in particular cytogenetics. Cohesin mutation were often observed in AML patients with t(8;21), but they were mutually exclusive in AML patients with favorable-risk cytogenetics, complex chromosomal abnormality, inv (16), and t(15;17) (11, 13, 37, 44–48), which also found in **Table 1**. Mutations in the gene of the cohesin complex rarely co-occurred with the spliceosome-complex, IDH1, IDH2, and TET2 and TP53 mutation (11, 13, 37). What is more, data from the Cancer Genome Atlas (TCGA) Research Network revealed that seldom AML patients carrying mutations in the cohesin complex had mutations in the IDH1 and IDH2 epigenetic modifiers, myeloid transcription factors, or NRAS (10).

Several studies have shown that mutations in the cohesin complex genes are often found in NPM1 mutation and CEBPAbi AML cases (11, 12, 37, 49, 50). Besides, lower expression of SMC1A was observed in patients with NPM1 mutation (51). But STAG2 mutation less frequently co-occurred with NPM1, and more frequently with RUNX1 (35, 37). It was worth noting that cohesin and NPM1 interact with the CCCTC-binding factor (CTCF) at the insulator sites through which they are involved in the insulator function (52, 53). Given the significant correlation between cohesin and NPM1 mutation, it is suggested they may have functional relevance in transcriptional regulation of the pathogenesis and mechanism in AML.

Contradictory results have been reported regarding the correlation between genes in the cohesin complex and other mutations. Mutations in Ras-family oncogenes including ASXL1 or BCOR, and FLT3-ITD were enriched in leukemia patients carrying the cohesin mutation (12, 37). However, No correlation was found between the cohesin complex and FLT3-ITD in AML (11).

## Clinical and Prognostic Implications of Cohesin Gene Mutations

The prognostic significance of cohesin mutations in AML remains controversial, and there are many factors that affect the prognostic significance of cohesin mutations. Tsai et al. suggested that cohesin mutations were independent favorable factors for a higher overall survival (OS) and disease-free survival (DFS) in *de novo* AML (37). Regardless of the cytogenetics, OS of adult AML patients harboring cohesin genes mutations tended to be higher than that of those without mutations (54). However, in contrast to these results, other studies found no survival differences were identified between mutant and wild cohesin in OS, DFS, and RFS at the different prognostic groups, such as total AML, pediatric AML, *de novo* AML (11, 12, 35, 54, 55). Despite cohesin gene mutation more frequently identified in sAML, CN-AML, and AML patients with NMP1 mutation, cohesin gene mutations had no significant implication on OS, RFS, and CR (11, 56). In addition, the outcome of allogeneic transplantation was not influenced by the presence of mutations in the cohesin complex (11). However, lower SMC3 protein levels were significantly associated with European Leukemia Net (ELN) adverse risk group classification (57). Thus the relationship between cohesin gene mutation and the prognostic is conflict.

Though there is a high frequency of cohesin gene mutation in t(8;21) AML, Faber *et al.* found that recurrent cohesin mutations did not affect the prognostic results of t(8;21) AML in 10 pediatric and 6 adult patients (58). Besides, AML patients with RAD21 mutation did not show statistical significance in OS and risk for relapse compared to those with wild RAD21 in the t(8;21) AML (13). However, recent works have shown that a mutation in cohesin genes was an independent poor prognostic factor for RFS and OS in patients with RUNX1-RUNX1T1 leukemia (43, 46). Among patients who had t(8;21) AML with tyrosine kinase (TK) mutations, those who had co-occurrent mutations in cohesin genes also had worse prognosis with a high 5-year cumulative incidence of relapse (CIR) and a higher risk of relapse (44). This result indicates that mutated genes function cooperatively in these events.

These findings all emphasize that cohesin have different functions under various clinical features in the biological mechanism. Taken together, the AML subtype, karyotype, age, and co-mutated genes should be considered together with cohesin mutation as prognostic factors. In particular, it is worth evaluating the prognostic significance of cohesin mutations in t(8;21) AML.

## Role of Cohesin Mutation in the Evolution of AML

Previous studies indicated that the occurrence of mutations in cohesin genes was considered as pre-leukemic and early genetic events in human leukemogenesis (18, 59, 60). During the progression from refractory anemia with excess blasts (RAEB) to sAML, cohesin mutations were observed only in patients with sAML, which implied that the occurrence of cohesin mutations represented a late event in the process of AML transformation (12). Mutations in SMC1A, SMC3 and STAG2 might be the initiating events in the pathogenesis of AML (55, 61). STAG2 mutation was identified as a secondary-type mutation in secondary and *de novo* AML (35). In addition, STAG2 mutation was found to occur in the early stage of AML, whereas RAD21 and SMC1A mutations occurred relatively late in adult AML (37). In the clonal architecture of t(8;21) AML, SMC3, and SMC1A mutations occurred at diagnosis, while RAD21 and STAG2 mutations appeared at relapse (13, 45). Hence, more systematic investigations are needed to decipher the evolution of cohesin gene mutations in AML.

## Mechanisms of Cohesin Gene Mutations in Leukemogenesis

Mutated cohesin complex genes are almost mutually exclusive (34), suggesting the changes in one subunit may affect the entire complex function. Interestingly, gene expression in the entire cohesin complex was usually decreased in AML patients with cohesin mutations (12). Normal sister chromatid cohesion was found in AML patients with cohesin mutations. Moreover, no significant difference was found in the number of chromosome abnormalities between cohesin-mutated and non-mutated patients (34). Genomic stability was not affected when cohesin levels were reduced by 80% in yeast and Drosophila embryos (62, 63). Nevertheless, no correlation between was found transcription and translation of cohesin subunits was found in 48 AML patients analyzed in a quantitative manner (57).

A lower protein level of SMC3 was found to be associated with numerical chromosome abnormalities and complex karyotypes (57). In mice, bone marrow aplasia with premature sister chromatid separation was caused by biallelic loss of SMC3 and chromatin structure and hematopoietic stem cell (HSC) function were controlled by cohesin in a dose-dependent manner (64). Given the above information, some cohesin mutations may have primarily affected its repair function and could not be selected clonally, leading to chromosomal instability and aneuploidy. Mitotic disorder or cell death occurred due to complete loss of the cohesin function, thus the majority of cohesin mutations were heterozygous in AML. Though genes encoding STAG2 and SMC1A were located on the X-chromosome, the homologous protein may be partially compensated for the loss of STAG2 function caused by its alterations (65, 66). Given the results of other study, SMC1A mutations are neither significantly associated with X loss nor with gender in CBF leukemia patients (46), which indicated that homologous protein of SMC1A might exists in leukemia patients.

In early hematopoiesis and myeloid differentiation, self-renewal of hematopoietic stem and progenitor cells (HSPCs) was enhanced, and differentiation of HSPCs was impaired as a result of cohesin mutation or deletion (60, 66–68). STAG2 loss decreased the transcription of lineage-specification genes, thereby leading to an altered hematopoietic function in a mouse model (66). However, STAG2 knockdown caused an increase in HSC-specific genes in primary human CD34+ cells (67). In HSPCs with the cohesin complex mutation, knockdown of ERG, GATA2, or RUNX1 can revert the differentiation block caused by cohesin mutation (69). The cohesin complex directly recruits and/or stabilizes the binding of Polycomb Repressive Complex 1 (PRC1) to active genes (70). The expression of self-renewal genes Hoxa7 and Hoxa9 in HSPCs increased through derepression of Polycomb Repressive Complex 2 (PRC2) caused by depletion of the cohesin subunit Rad21 (71). In addition, cohesin deficiency in hematopoietic progenitor cells could not induce acute phenotypes through a series of shRNA mouse models (60). Self-renewal capacity of HSPCs increased as a result of SMC3 haploinsufficiency, but SMC3 knockdown together with FLT3-ITD could lead to the development of AML (64). Thus, heterozygous mutations may depend on the cooperation with other mutations for causing leukemia.

Previous studies suggested that some hematopoietic transcription factors such as TAL1 and ERG, are regulated by cohesin binding to distal enhancers or promoters (72–75). A recent research has shown that *Stag2* and *Runx1* play an important role in regulating chromatin looping and transcription in hematopoiesis, and cohesin-Runx1 deficiency can induce myeloid-skewed expansion of HPSCs and myelodysplastic syndromes (MDS) (76). In mouse and zebrafish, cohesin binds to *runx1* enhancer region, which is located within the intron between the P1 (distal) and P2 (proximal) promoters of *Runx1* (32, 77, 78). Similarly, cohesin subunits are found in *conserved* regulatory elements (CRE)/promoter of *Runx1* in the human leukemia K562 cell line (32). It plays an indispensable role in terms of regulating *Runx1* expression in a specific subpopulation of hematopoietic progenitors and cohesin together with CTCF determines the spatial distribution of runx1 transcripts in the zebrafish embryo (32, 79). Monoallelic loss of *Rad21* resulted in a decrease in the transcription of *runx1* and micro-injection of *runx1* mRNA can rescue the defects caused by RAD21 mutations in differentiated blood cells of the zebrafish model (80). Nevertheless, RNAi-mediated cohesin knockdown was found to enhance RUNX1 transcription in the leukemia HL60 cell line (32). Moreover, Mazzola *et al.* found there was no significant correlation between the expression of RAD21 and RUNX1 in both megakaryocytes derived from healthy donors and megakaryocytes derived from adult AML patients (80). These evidences indicate that cohesin performs different functions in regulating RUNX1 expression in different species.

**Figure 3:**
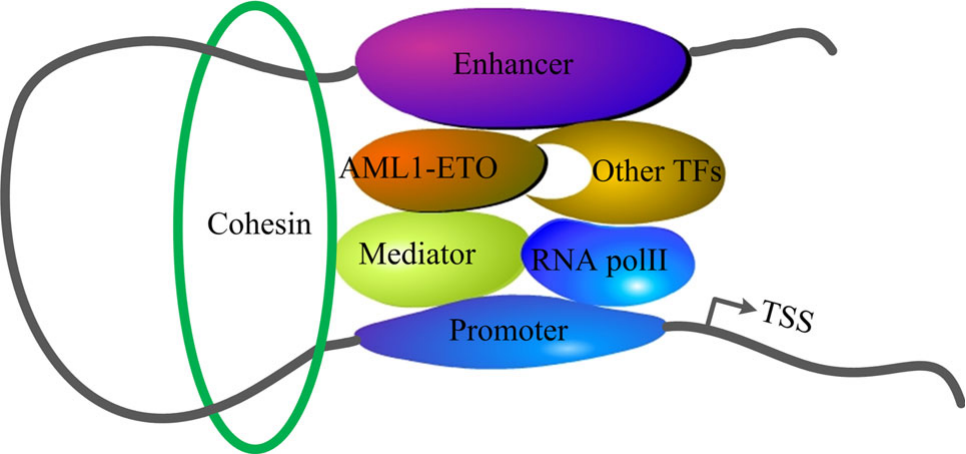
Interactions of cohesin and AML1-ETO.

Cohesin mutations were prevalent in AML with RUNX1 abnormality (12), especially in RUNX1-RUX1T1. Significant growth suppression of the Kasumi-1 cell (harboring t(8;21) and RAD21 frameshift mutation p.K330PfsX6) was induced by forced expression of wild-type RAD21 (34). These results suggest that there is potential synergy between cohesin and AML1-ETO in hematopoiesis and leukemogenesis. Furthermore, AML patients with cohesin mutations expressed significantly higher levels of AE9a than those without cohesin mutations (81). AML1-ETO blind to the promoter and distal elements including those of itself, and it interacts with other hematopoietic transcription factors, such as ERG, FLI1, TAL1, and RUNX1, in this region (82). Importantly, the promoter can interact with short or long distances enhancers to activate gene expression, independent of the position and orientation (83). Given the fact that TFs are mainly enriched in enhancers to control gene expression (84), cohesin can eliminate the spatial distance of promoter and enhancer by promoting chromatin loop formation (85–87). Therefore, we postulate that AML1-ETO recruits hematopoietic TFs to the enhancer. Then, the mediator complex and RNA polymerase II (Pol II) blind to the TFs and promoters respectively, in a gene-specific manner. Cohesin is loaded onto the promoter and enhancer that contribute to gene regulation (**Figure 3**). RUNX1 and AML1-ETO share the same sequences before exon 5; thus, cohesin may also be involved in the regulation of AML1-ETO expression.

## Conclusion and Outlook

In this review, we summarized and analyzed cohesin mutations in AML, especially in t(8; 21) subtype, from previous studies. The conclusion and outlook are as follows:

1. We found each subunit of cohesin mutated in different characteristics with different percentage. RAD21, SMC1A, and SMC3 mutations were remarkably higher in t(8; 21) than the other AML subtypes. STAG2 mutation occurred chiefly on adult AML and sAML. For FAB subtype, RAD21 and SMC3 are mainly found in FAB M2. SMC1A was common found in male. Such differences arise because each individual member of the cohesin complex has different influence and function in different characteristics of AML by different ways and mechanisms.
2. Most of the current studies confirmed that cohesin can act as a growth regulator of HSCs. Whether cohesin gene mutation leads to protein functional activation or inactivation is unclear. Although mutated cohesin perturbed the balance between self-renewal and differentiation, no evidence demonstrated that the impaired cohesin function could independently lead to AML, suggesting that cooperative genetic/epigenetic alterations are involved in AML development. So far, co-mutations of cohesin with other genes are still controversial and the biological functions of cohesin are not yet fully understood in AML.
3. The clinical outcomes of cohesin mutations are associated with the AML type, karyotype, and co-mutated gene. No specific therapeutic strategies targeting cohesin mutated AML have been described. In addition, most of the studies indicated that cohesin mutations are pre-leukemic and early genetic events in human leukemogenesis. Considering the four core subunits of cohesin associated with different prognosis and mutation frequencies, their specific functions in AML should be also investigated in depth.
4. The mechanism with respect to cohesion subunits mutation is still unestablished in AML, especially in the t(8; 21) AML. How cohesin subunits mutation cooperates with AML1-ETO, thereby promoting leukemic transformation should be illustrated in future studies. Further understanding of the relationship between cohesin and AML1-ETO may provide unprecedented insights into the pathogenesis of AML.

Conducting research on above questions would hopefully provide significant progress in diagnosis classification, prognostic stratification, and therapeutic strategies to determine AML risks in the near future.

## Author Contributions

CH wrote the manuscript. XG and YL revised the manuscript critically. JZ, EY, and LZ prepared the figures and revisions. LY contributed to study design, writing, and editing. All authors contributed to the article and approved the submitted version.

## Funding

This work was supported by “Major New Drug Development Project” from Ministry of Science and Technology of China (2019ZX09201002003), Natural Science Foundation of Shenzhen University General Hospital (SUGH2020QD008), State Key Program of National Natural Science of China (82030076), National Natural Science Foundation of China (82070161, 81970151, 81870134, and 82000161), Beijing Natural Science Foundation (7202186), Shenzhen Science and Technology investigation project (JCYJ 20190808123005569 and JCYJ 20190808163601776), China Postdoctoral Science Foundation (2018M640824), and Science and Technology Foundation of Shenzhen (JCYJ20200109113810154).

## Conflict of Interest

The authors declare that the research was conducted in the absence of any commercial or financial relationships that could be construed as a potential conflict of interest.
